# Multilingualism and strategic planning for HIV/AIDS-related health care and communication

**DOI:** 10.12688/wellcomeopenres.15584.1

**Published:** 2019-12-12

**Authors:** Kathryn Batchelor, Lalbila Aristide Yoda, Féridjou Emilie Georgette Sanon Ouattara, Olivia Hellewell

**Affiliations:** 1 Centre for Translation Studies (CenTraS), University College London, London, WC1E 6BT, UK; 2 Université Ouaga I Professeur Joesph Ki-Zerbo, Ouagadougou, Burkina Faso; 3 Department of Modern Languages and Cultures, University of Nottingham, Nottingham, NG72RD, UK

**Keywords:** translation, interpreting, languages, health communication, development, HIV/AIDS, language barriers, West Africa

## Abstract

**Background: **Many lower and middle income countries (LMICs) have high levels of linguistic diversity, meaning that health information and care is not available in the languages spoken by the majority of the population. This research investigates the extent to which language needs are taken into account in planning for HIV/AIDS-related health communication in development contexts.

**Methods: **We analysed all HIV/AIDS-related policy documents and reports available via the websites of the Department for International Development UK, The Global Fund, and the Ministries of Health and National AIDS commissions of Burkina Faso, Ghana and Senegal. We used quantitative and qualitative analysis to assess the level of prominence given to language issues, ascertain the level at which mentions occur (donor/funder/national government or commission), and identify the concrete plans for interlingual communication cited in the documents.

**Results: **Of the 314 documents analysed, 35 mention language or translation, but the majority of the mentions are made in passing or in the context of providing background socio-cultural information, the implications of which are not explored. At donor level (DFID), no mentions of language issues were found. Only eight of the documents (2.5%) outline concrete actions for addressing multilingualism in HIV/AIDS-related health communication. These are limited to staff training for sign language, and the production of multilingual resources for large-scale sensitization campaigns.

**Conclusions: **The visibility of language needs in formal planning and reporting in the context of HIV/AIDS-related health care is extremely low. Whilst this low visibility should not be equated to a complete absence of translation or interpreting activity on the ground, it is likely to result in insufficient resources being dedicated to addressing language barriers. Further research is needed to fully understand the ramifications of the low prominence given to questions of language, not least in relation to its impact on gender equality.

## Introduction

Effective communication has long been recognised as a crucial factor in health care. Much of the focus of both academic scholarship and training for practitioners has been on intralingual communication, i.e. communication between individuals using the same actual language (English, French, etc.). Scholars have investigated intralingual communication across a wide variety of settings and channels including primary care, telemedicine, media campaigns, online health information, and everyday interpersonal communication ( Thompson *et al.*, 2011). There has also been some interest in interlingual communication, i.e. communication between individuals using different actual languages. The bulk of research in this latter area has been on migrant communities accessing health care in Western settings, notably people with limited English proficiency (LEP) accessing health care in the United States (see, for example, Brach *et al.* (2005); Elderkin-Thompson *et al.* (2001); Flores, 2005; Jacobs *et al.* (2004)). 

This research has found that language barriers can have a major adverse impact on health and health care ( Flores, 2005) and that people with LEP ‘have worse access to care, receive poorer quality of care, are less likely to understand and adhere to care plans and are less satisfied with their physicians’ ( Gregg & Saha, 2007, p. 368). Language barriers have also been associated with additional costs ( Hampers & McNulty, 2002), since LEP patients have more diagnostic tests ( Brach *et al.*, 2005).

Recent insights into the need for ‘transformative’ as opposed to ‘technical’ health communication ( Campbell & Scott, 2012, p. 179) reinforces the significance of language: in such transformative approaches, it is necessary not only for the patient to understand the information that is provided by the practitioner, but for the practitioner to understand the patient and to adopt approaches to health communication that are anchored in communities’ own analyses of their health needs and problems.

Despite the attested connections between language barriers and effective health care provision, there has been limited research into this issue in parts of the world where language barriers are most acute, in the sense of affecting the largest proportions of a country’s population. Many African countries, for example, have extremely high levels of linguistic diversity ^1^, with the number of languages spoken within each country ranging from around eight to around 275 ( Ethnologue). Although not all scholars agree on these statistics ^2^, it is clear that when health care provision is made available only in the official language(s) of the country concerned – usually a single language, and predominantly English or French – then large sections of the population find themselves excluded from good quality health information and unable to be active participants in their own health care, whether at an individual or community level. Whilst individuals – and still less communities – in African countries are rarely, if ever, monolingual, we should note that African multilingualism is ‘predominantly oral’ ( Adejunmobi, 2013, p. 25). This adds to the complexity of the situation, meaning for example that there may be no agreed-upon terminology for many medical conditions or treatments in many of the commonly used languages, or that individuals may not be literate in the languages which they speak or understand most easily.

In the African context, the bulk of scholarly enquiry into language barriers in health care has focussed on South Africa (see e.g. Deumert (2010); Levin (2006); Schlemmer & Mash (2006); van den Berg (2016)) or on the specific case of the Ebola outbreak in West Africa in 2014–15 (e.g. Journal of Health Communication supplement 22, 2017). Studies addressing other contexts or other parts of the African continent are far fewer in number (though for examples, see Acquah & Beck (2013); Anita & Bertin (2004); Diop (2016); Sanon Ouattara (2016); Yoda, (2007)).

The need for further research focussing on African contexts, particularly beyond South Africa, is pressing. Such research will allow us not only to redress the balance of scholarship, which is currently skewed towards Western contexts ( Kim *et al.*, 2010), but will also permit a re-envisioning of policy-making to the advantage of those who are currently excluded from health information or health care as a result of language barriers. Since the languages in which health information is exchanged are often those which are acquired via the education system rather than in family settings, addressing policy-making in this regard is also a means of addressing gender inequalities: globally, Sub-Saharan Africa ranks last on the Educational Attainment subindex of the Global Gender Gap, with four of the ten lowest-ranked countries on the literacy rate indicator being from this region ( World Economic Forum, 2017, p. 23). This means that women are more likely to be unable to access quality health information when information is provided only in official languages. At the same time, it is women who are disproportionately affected by HIV/AIDS in the Sub-Saharan African region, to the extent that official documents now speak of a ‘féminisation de l’épidémie’ [feminisation of the epidemic] ( CNLS Senegal, 2006, p. 24). Improving communication with women and girls is of paramount importance to efforts to curb infection rates, prevent mother-to-child transmission, and improve the outcomes for those already living with the disease. 

## Research questions

This study seeks to assess the extent to which linguistic diversity is taken into account in strategic planning for HIV/AIDS health care and communication in development contexts, with a particular focus on West Africa. Where multiple actors are involved, it also aims to identify the level at which strategic planning takes place (donor government, funder, national government, national AIDS commission). Finally, the study summarises the specific means of addressing language barriers that are explicitly outlined in strategic planning documents and reports.

## Research design

### Scope and limitations

The organisations and groups that are involved in strategic planning for HIV/AIDS health care and communication in West Africa can be broken down into four broad categories:

- Donors (e.g. Department for International Development (DFID), European Commission)
- Funders (e.g. Global Fund, World Bank)
- West African governments and national HIV/AIDS commissions (e.g. Ghana Ministry of Health, Ghana Aids Commission)
- Organisations working in West African countries (non-government organisations (NGOs), civil society organisations, charities, private sector organisations, etc.)

According to the UNAIDS strategy 2016–21 ( UNAIDS, 2016, p. 102), West Africa relies on international funding for 70% of its funds to fight AIDS, so while in some cases strategic planning might be the preserve of the third and fourth categories alone, in the majority of cases it will be relevant to consider the interplay across all four categories. 

While the potential number of organisations spanning these four categories in the 16 countries of West Africa is high, for the purposes of this desk-based research article the scope is restricted to an analysis of one organisation from each of the first two categories, and to three West African government departments with their associated government-led AIDS commissions from the third. The limited internet presence of many of the organisations in the fourth category requires a different set of research methods and merits a separate investigation. The organisations selected for inclusion in the corpus are presented below, together with the rationale for their selection.

### Donor organisation: DFID

The UK’s DFID represents one of the largest government donors to multilateral funding organisations including The Global Fund ( The Global Fund, 2019a), and is also a significant bilateral official development assistance (ODA) provider to African countries ^3^. The rationale for its inclusion in this study thus lies in its significance as a global player in the fight against HIV/AIDS: the strategic plans made by DFID not only have direct implications for African countries which receive its bilateral funds, but also potentially influence the strategies adopted by the large funding organisations ^4^.

### Funder: The Global Fund

The Global Fund is one of the world’s three largest AIDS donors ( Oomman *et al.*, 2007), and tends to be the major donor in the West and Central Africa region ( Avert, 2018). The Global Fund is the principal mechanism used by DFID to finance their contribution to HIV and tuberculosis (TB) treatment ( Department for International Development, 2011, p. 12). The rationale for its inclusion in this study thus lies like DFID’s in its significance as a donor and major strategic planner in the fight against HIV/AIDS.

### Ministries of Health and National AIDS Commissions in Burkina Faso, Ghana, Senegal

Burkina Faso, Ghana, and Senegal were selected for this study on the basis of the key similarities that they share, notably with regard to linguistic diversity indices (0.721, 0.858, 0.778, respectively; see Ethnologue) and per capita spending on health (4–6%) ( Global Burden of Disease Health Financing Collaborator Network, 2018, pp. 1803–1810). These similarities allow us to hypothesise that our findings will be likely to obtain for other countries in the West African region or even for low-income countries with high linguistic diversity elsewhere in the world. At the same time, the similarities mask important differences. With regard to linguistic diversity, Senegal is the only country of the three (and indeed, the only country in the West African region), to have a national language (Wolof) which has emerged as a national lingua franca and which has come to rival the official language (French) in terms of its use in institutional and educational contexts ( McLaughlin, 2008). There are also differences between the three countries in the official language (English in Ghana; French in Senegal and Burkina) and in estimates of the number of proficient speakers of that official language (22% in Burkina and 29% in Senegal ( Organisation Internationale de la Francophonie, 2014, p. 17); 61.7% in Ghana ( ( Ghana Statistical Service, 2012, p. 6)), as well as in overall literacy rates (36%, 71%, 56% respectively; see Ethnologue). In terms of overall wealth, we should note that Ghana’s GDP is more than four times that of Burkina and nearly three times that of Senegal ( World Bank, 2018). There are also differences between the three countries in terms of the levels of national government funding allocated to HIV/AIDS spending and the amount of developmental assistance for HIV/AIDS spending received in relation to that state’s total HIV/AIDS spending ( Burden of Disease Health Financing Collaborator Network, 2018), and slight differences in HIV prevalence rates (0.8% in Burkina, 1.7% in Ghana, 0.4% in Senegal; see UNAIDS). Any of these factors might serve as explanations for differences that may be found in our study, and our research design means that we are not able to isolate one from the other. Nevertheless, the emphasis on qualitative rather than solely quantitative analysis in our study allows us to work inductively to develop further hypotheses which can be tested in subsequent studies. 

For each of the three countries, we analysed documents produced both by the Ministry of Health and by the national HIV/AIDS commission which operates under the office of the President in each case. The rationale behind including both types of government organisation is that both are involved in strategic planning pertinent to HIV/AIDS communication, the former primarily in the shape of overarching health policy, and the latter with regard specifically to strategies for targeting HIV/AIDS.

## Research methods

### Selection of documents for analysis

Documents were selected for analysis following principles of systematicity and comprehensiveness. For each of the organisations or government departments in question, we used their official websites to search for all documents relevant to HIV/AIDS. We limited the scope only by document type, restricting our corpus to policy documents (including funding guidelines) and reports, rather than encompassing a broader range of documents such as press releases or research papers. We did not limit the searches by date, including in the corpus any policy documents or reports which the organisation or government department in question made available via their website during the periods in which we gathered our data (November-December 2018 and May-July 2019). In cases where the government website was not functioning, we identified relevant documents through step-by-step processes which are described in detail in the sub-sections below.

### DFID

The DFID documents were identified using the UK government website https://www.gov.uk/search/all using the following parameters: Search term: ‘HIV’; Topics: all; Sub-topics: all; Show only: ‘Guidance and Regulation’; ‘Policy papers and consultations’; Organisation: ‘DFID’. The search was carried out on 8 July 2019 and yielded 35 results. Of these, six were excluded because their primary focus was on topics other than health (e.g. ‘Social Dimensions of Transport’) or were not authored by DFID (e.g. pledges made by other organisations to sign up to summit commitments). Some of the results contained child pages, meaning that 66 documents were identified in total through the government website search. One further document, the ‘UK Aid Strategy’, also available on the gov.uk website, was added to the corpus for the reason that all DFID spending is carried out in line with this strategy.

### The Global Fund

The Global Fund website was interrogated between 1 May 2019 and 15 June 2019, and the following documents were selected for inclusion in the corpus:

- All documents on the ‘Publications’ page.
- All documents on the ‘Country Coordinating Mechanism’ (CCM) page.
- All documents on the ‘Funding Guidelines’ page.
- All documents pertaining to the three countries under study: Burkina Faso, Ghana, Senegal. These documents were identified via two means: 1) by setting the country filter to the relevant country on the ‘ Find a Grant’ page; 2) via the ‘Overview’ pages for Burkina Faso, Ghana and Senegal.

The rationale for selecting these documents were as follows: the documents listed on the ‘Publications’ page can be argued to reflect the key concerns of the Fund and to encompass the most up to date and important strategy documents; the CCMs play a key role in determining the specific strategies used to combat the epidemics; the Funding Guidelines are used by applicants to formulate their requests for funding, and as such shape the projects undertaken; country-specific documentation speaks to the agendas and priorities set by the countries themselves.

The total number of documents selected in this way came to 182. From this initial corpus, any documents which were purely technical (e.g. specimen signature templates), pertained exclusively to topics or regions not relevant to our research or which were shorter summaries of other documents were excluded. After these exclusions, the total number of documents selected for analysis came to 150.

### Burkina Faso: Ministry of Health

We included all policy documents and reports available on the Burkina Faso Ministry of Health website, excluding from our corpus only those documents which were specific to illnesses other than HIV/AIDS or which were shorter summaries of other available documents. The date of the interrogation of the website was 10 December 2018. One of the documents found on the website (‘Plan de développement sanitaire’) pertained to the period 2001–2010, and we therefore used the name of the report as a search term in Google to find the current version of the policy. A further two documents were identified via the Google results generated by this search.

### Bukina Faso: Conseil National de Lutte contre le SIDA (CNLS)

The website of the CNLS (National Council for the Fight against AIDS) was not functioning during either of the periods in which we carried out research for this article. Policy documents and reports authored by the CNLS were therefore found via Google searches using the following general search terms: ‘burkina faso politique vih’ [Burkina Faso HIV policy], ‘CNLS Burkina filetype:pdf’. We also used Google to search for specific documents mentioned in reports or news articles, notably the ‘Cadre stratégique national de lutte contre le sida 2016–2020 Burkina Faso’ (mentioned on the news website iBurkina (2018)), and the ‘Plan d’urgence pour l’accélération de la réponse nationale au VIH 2017-2018’ (mentioned in a UNICEF press release ( UNICEF, 2017)). While we did not succeed in finding either of these recent documents, the Google searches did lead to the identification of three further CNLS policy documents.

### Ghana: Ministry of Health

We included in the corpus all documents available via the ‘policy documents’ and ‘annual reviews’ sections of the Publications tab of the Ministry of Health website with the exception of three documents dealing with topics not relevant to this research (e.g. ‘Anti-Malaria Drug Policy’). The date of the interrogation of the website was 3 December 2018.

### Ghana: Ghana Aids Commission (GAC)

The Publications page of the Ghana Aids Commission website does not differentiate documents by category. We therefore evaluated each of the 55 documents available on the site, selecting for inclusion in the corpus those which could be classified as policy documents or reports (17 documents in total). The categories of items thus excluded encompassed data management manuals, press releases, and terms of reference documentation. We also excluded abridged versions of documents already included in the corpus. The date of interrogation of the website was 10 December 2018.

### Senegal: Ministry of Health

As in the case of the GAC, the Senegal Ministry of Health website does not differentiate documents by category. Once again, we therefore assessed all documents for their relevance to the project, carrying out our website searches between 15–25 May 2019. The vast majority of documents were of a type or on a topic unrelated to our research project (e.g. legal documents, parliamentary bills, instructions on requesting health-related certificates). In total we found eight documents which could be categorised as policy documents or reports. In addition, we used Google to search for the ‘Plan national de développement sanitaire (PNDS)’, a document mentioned on the Ministry of Health website, but not available on the website itself. The Google search yielded two iterations of this plan, PNDS 2004–2008, and PNDS 2009–2018, as well as three further relevant policy documents produced by the Senegalese Ministry of Health.

### Senegal: Conseil national de lutte contre le SIDA (CNLS)

The CNLS website hosts a comprehensive set of policy documents and reports on the ‘Bibliothèque’ tab. The publications are grouped under eight different headings, three of which were of relevance to our study (‘Plan Stratégique’ [Strategic Plan], ‘Rapports’ [Reports], ‘ENCS’ [National Monitoring Reports]). All of the documents available under these headings at the time of our interrogation of the website (15–25 May 2019) were included in our corpus. The strategic plans covered the years 2002–2022, the annual reports covered 2005–2009 and 2012–17, the national survey reports were dated 2006, 2010 and 2015, and the other documents were from 2018. There was therefore nothing significant missing from the documentation available on the CNLS website and we did not carry out any Google searches for this part of the study.

### Analysis of documents

The documents were analysed using a two-stage process. In the first stage, each document was searched for key terms pertaining to languages and translation using Acrobat Reader DC (version 2019.012.20034; for pdfs) or Microsoft Word 2016 (for Word documents). Where automated searches did not work (for example, with some scanned pdf documents), the documents were searched manually. For documents in English, the following search terms were used: ‘language’; ‘lingu’ (to capture ‘multilingual’ ‘polylingual’, ‘linguistic’, ‘multilingualism’, and so on); ‘translat’ (to capture ‘translation’, ‘translator’, ‘translated’). For documents in French, the following equivalent search terms were used: ‘langue’; ‘lingu’ (‘multilingue’, ‘polylingue’, ‘linguistique’, ‘multilinguisme’ etc.); ‘tradu’ (‘traduction’, ‘traducteur’, ‘traduit’). To ensure that no mentions were missed, each document was also searched for the term ‘communication’ (in French or English), and for potentially relevant named languages (e.g. ‘English’, ‘French’, ‘Wolof’, ‘Moore’, ‘Twi’).

In the second stage, each hit and its textual context was subject to close reading to ensure the pertinence of the hit for the quantitative part of the study and to generate findings for the qualitative aspect of the study. Close reading was particularly important for guaranteeing the accuracy of the quantitative results, since ‘translate’ or ‘traduire’ and their variants are frequently used in both English and French in metaphorical senses rather than to denote translation between languages. For example, in the Global Fund corpus, ‘translation’ is used in the sense of translation between currencies ( 2018 Annual Financial Report), translating ideas into practice ( Gender Equality Strategy Action Plan 2014–2016), translating issues into investment ( Gender Equality and Key Populations), or translating funding requests into disbursement-ready grants ( Operational Policy Manual).

‘Language’ is another term with a broader set of possible meanings than our intended sense of denoting one of the languages spoken in the world. In the corpus as a whole, close reading showed that many of the hits for language denoted intralingual language use, and these were therefore discounted. For example, in the ‘Strategic Investments for Adolescents in HIB, Tuberculosis and Malaria Programs Information Note’ ( The Global Fund, 2016a, p. 15), information is to be provided ‘in language that is understandable to adolescents’. This is clearly a reference to age-appropriate language rather than to a particular language such as French or Wolof.

## Results

In total, 314 documents were analysed (see extended data ( Batchelor *et al.*, 2019) for a full list of documents). The number of documents that contain hits for one or more of the search terms identified above and were deemed through close reading to have mentioned the issue of language use in the sense pertinent to our research question is 35. This equates to 11.1% of the documents. As we will see below, the majority of these mentions are extremely brief.

There are significant differences in the percentage rates for each organisation or government department, as shown in Table 1. Both DFID and the Senegalese Ministry of Health have zero mentions of language or translation. The next lowest in terms of percentage of mentions is the Global Fund. The organisation with the highest percentage of mentions is the Burkina Faso CNLS. Of the three countries studied, Burkina has the highest mention rates (33.3% and 50%), while Senegal has the lowest (0% and 20%). In the case of all three of the countries, the national HIV/AIDS commission is more likely to mention language than the corresponding government Ministry of Health.

**Table 1.** Number of HIV-related policy documents/reports which mention “language” or “translation” in the sense of addressing language barriers. The data was generated by searching the organisations’ official websites between November 2018 and July 2019 and was supplemented by Google searches where websites were not functioning or obviously contained incomplete documentation records.

Organisation	Number of documents analysed	Number of documents that mention “language” or “translation”	Percentage of documents that mention “language” or “translation”
UK Department for International Development (DFID)	67	0	0%
Global Fund	150	14	9.33%
Burkina Faso Ministry of Health	9	3	33.3%
Burkina Faso Conseil National de Lutte contre le SIDA (CNLS)	8	4	50%
Ghana Ministry of Health	30	4	13.33%
Ghana AIDS commission	17	6	35.29%
Senegal Ministry of Health	13	0	0%
Senegal Conseil National de Lutte contre le SIDA (CNLS)	20	4	20%

While these figures suggest that strategic planning for addressing language barriers is more likely to take place at country level rather than at donor or funder level, in themselves they are too bald to tell us much about the relative prominence given to language issues. They do not differentiate, for example, between background mentions of language on the one hand and sustained discussions of language barriers on the other. In some cases, they also risk being misleading: the four mentions in the Senegal CNLS corpus, for example, all occur in serial annual reports, with exactly the same wording being used on each occasion. Had the corpus only included the most recent annual report, then the percentage calculation would be markedly lower. For these reasons, the sub-sections that follow will present both a nuancing of these quantitative results and a qualitative analysis of the mentions found in the documents.

### Global Fund

The majority of the mentions of language in the Global Fund corpus are cursory. For example, the only mention of translation in the 24-page ‘Global Fund Gender Equality Strategy’ is found in an annex which outlines opportunities for partner engagement: it is noted that ‘partners can also provide transport, translation, capacity building and training’ ( Gender Equality Strategy, p. 20). Similarly, in the ‘Strategy in Relation to Sexual Orientation and Gender Identities’, it is noted that partners might support ‘conducive national environments’ by ‘translating tools and resources’ ( Strategy in Relation to Sexual Orientation and Gender Identities, p. 20), and ‘translation’ is also listed as an example of ‘communication costs’ that can be covered by Global Fund supplemental administrative funding for Country Coordinating Mechanisms (CCMs) (Strategy in Relation to Sexual Orientation and Gender Identities, p. 13).

The Global Fund website lists policy documents and reports under ten different categories. We found significant variations in the number of mentions of language under each of these categories (see Table 2).

**Table 2.** Number of HIV-related policy documents/reports on The Global Fund website which mention “language” or “translation” in the sense of addressing language barriers. The data was gathered between 1 May 2019 and 15 June 2019 and is ordered by the categories used on the Global Fund website.

Document Category	Number of documents analysed	Number of documents which mention “language” or “translation”
‘About the Global Fund’	9	0
Community, Rights, Gender	14	6
‘Focus on’ Reports	11	0
Impact Reports	3	0
Donor Reports	15	1
Country Coordinating Mechanism	8	2
Funding Guidelines	25	4
Country-specific documents: Burkina	19	1
Country-specific documents: Ghana	27	0
Country-specific documents: Senegal	19	0
**Totals**	**150**	**14**

Table 2 shows that the language issue is accorded greatest attention in the group of documents subsumed under the heading ‘Community, Rights, Gender’. The topics with which the documents under this heading deal are community participation, human rights and gender equality, and concern in particular the Global Fund’s efforts to ensure that its country dialogue processes are fully inclusive. The tendency for language to be mentioned in connection with country dialogue processes is also found in the documents belonging to the other categories: in all, 20 out of the 25 mentions of language that occur in the 14 Global Fund documents concern community participation in Global Fund agenda- and priority-setting processes. Only five of the mentions of language (occurring across three different documents) address language barriers in the context of health care or health communication activities themselves.

The overall level of attention accorded to questions of interlingual health communication is thus extremely low, and the multilingual nature of many of the environments in which the Global Fund operates emerges only in connection with the Global Fund’s operating principles rather than in connection with the actual health care and communication activities that are made possible through the Fund. Indeed, one of the most striking aspects of the Global Fund results is the near-total absence of mentions of language in documentation that is produced for the Global Fund by the three countries under study. Of the 65 documents in this category – which consist primarily of funding proposals – only one mentions language. This document, the ‘Burkina Faso Proposal to the Global Fund HIV/AIDS Sixth Call (2006)’, identifies ‘large scale sensitization campaigns and targeted advertisement in local languages’ ( Burkina Faso CCM, 2006, p. 69) as one of the means through which the country will try and fight against HIV/AIDS stigmatization. There are no similar mentions of multilingual campaigns in any of the other funding proposals put forward by Burkina or in any of the proposals put forward by Ghana or Senegal, these proposals preferring instead to speak for example of ‘listening and interpersonal communication activities’ ( Burkina Faso CCM, 2010, p. 19) or ‘communication materials’ ( Adventist Development and Relief Agency (ADRA) of Ghana, 2015, p. 22). The use of the hypernym ‘communication’ is an issue to which we will return in the Discussion section. 

### Burkina Faso Ministry of Health

Of the three Ministry of Health documents that mention language or translation, two of them do so only in terms of providing background socio-cultural information for the remainder of the report. Both the ‘Plan de développement sanitaire 2001–2010’ [Health Development Plan 2001–2010] and the ‘Document de politique sanitaire nationale’ [National Health Policy Document] include identical introductory paragraphs about Burkina Faso, noting that the population is made up of ‘une soixantaine de groupes ethnolinguistiques’ ( Burkina Faso Ministry of Health, 2001, p. 8) [approximately sixty ethnolinguistic groups] and that ‘les principales langues parlées sont le moore, le dioula et le fulfulde’ ( Burkina Faso Ministry of Health, 2001, p. 8) [the most widely spoken languages are Moore, Dioula and Fulfulde]. Languages find no further mention in these relatively lengthy reports (64 pages and 31 pages respectively), and the implications of this information are not spelt out in any way.

The third document once again has a single mention of language, but this time language is explicitly connected with health communication initiatives. In the ‘Profil sanitaire complet du Burkina Faso 2015’ [Burkina Faso Full Health Profile 2015], a 73-page report jointly produced by the Ministry of Health and the World Health Organisation in Burkina Faso, the list of communication activities undertaken in 2013–15 includes ‘émissions radios et télé en langues nationales, confection/ diffusion de spots radio/télé en français et en langues nationales’ ( Burkina Faso Ministry of Health, 2017, p. 35) [radio and TV programmes in national languages, production/diffusion of radio/TV adverts in French and national languages]. Strictly speaking, this mention should not count towards the quantitative findings, as the focus at this point in the report is on vaccination campaigns rather than HIV/AIDS. As many of the documents in the corpus as a whole deal with health in a broader way, We have not discounted it, but the key point to note here is that what looks statistically like a considerable amount of attention being paid to language issues by the Burkina Ministry of Health (33.3% of documents) in fact breaks down into two passing mentions and one non-HIV/AIDS-related mention. Even where a language-related initiative is outlined, no detail is provided on the number of languages in which material was produced (the Ethnologue gives the number of living languages in Burkina as 71) or how audiences who do not speak those languages would be reached through the campaigns. Language, in other words, is accorded barely any explicit attention.

### Burkina Faso CNLS

Of the four Burkina Faso CNLS documents which mention language, three do so in very brief terms. The ‘Global Aids Reporting (GARP)’ 2012 report mentions language in the context of a case study of women working as casual vendors, noting their low education levels and suggesting the ‘barrière linguistique’ [language barrier] ( Burkina Faso CNLS, 2012, p. 39) as one of four possible reasons why so few women in this socio-economic category have taken part in Information Education Communication (IEC) activities. The report does not elaborate on what is meant by the language barrier or explain the connection between education levels and language competencies.

An even more fleeting mention is found in the ‘Cadre stratégique de lutte contre le VIH, le SIDA et les infections sexuellement transmissibles’ (2011–2015) [Strategic Framework for the Fight against HIV, AIDS and sexually transmitted diseases (2011–2015)]. The report suggests that one of the reasons for the levels of protection against HIV being low among ‘les indigents’ [people living in poverty] is as follows:

l’inadéquation entre les sources d’information sur le VIH et les canaux par lesquels les indigents arrivent à recevoir les informations y relatives. En effet, au niveau national, les principales sources de l’information disponibles sont les supports écrits en français (manuel de formation, d’information, rapports divers, affiches etc.), alors que les indigents s’informent principalement par le biais de la radio et souhaitent recevoir toute information par ce même canal. ( Burkina Faso CNLS, 2011, p. 40).
[disparities between sources of information about HIV and the channels through which those living in poverty receive information about HIV. At the national level, the main sources of available information are written material in French (training and information manuals, reports, posters etc.), even though people living in poverty obtain information principally through radio and would rather receive information through this channel.]

The way in which this is phrased presents the problem primarily as one of communication channel rather than language, comparing written documentation in French with radio. What is left implicit is that radio communication often takes place in national languages, thanks to a strong network of local radio stations ( Capitant, 2008). The fact that the Strategic Framework does not tease apart language and communication channel is an indication of the extent to which questions of language and literacy are interconnected in Burkina: the majority of the population is literate in French rather than in the national languages, and those who have not completed primary school are unlikely to speak French well.

The third document, the ‘Rapport sur le développement humain durable Burkina Faso 2001 - La lutte contre le VIH-SIDA’ [Report on sustainable human development Burkina Faso 2001 – The fight against HIV/AIDS], similarly conflates the question of language with the question of communication channel. In a thoughtful reflection on the cultural determinants of HIV/AIDS, the report notes that cultural aspects have not been integrated in the prevention process, and prevention campaigns have been organised ‘en langue française à la radio et à la télévision alors que seuls 15 à 20 % de la population sont alphabétisés et touchés par ces programmes de sensibilisation’ ( PNUD, 2001, p. 116) [in French on radio and TV even though only 15–20% of the population are literate and reached through these awareness campaigns]. The conflation of literacy and French language competence is particularly notable in this statement, as radio and television programmes are primarily oral channels of communication, and it is therefore not a lack of literacy that impedes comprehension for this group, but a lack of French language competence. The only other mention of language in this substantial 231-page report comes in one of the feature boxes that provide snapshots of relevant activities. This box features the case of the four-part educational documentary film ‘Faso contre le SIDA’ [Faso against AIDS] and notes that 400 copies of the film were produced ‘couvrant chacune des quatre principales langues nationales’ ( PNUD, 2001, p. 89) [in each of the four main national languages].

The fourth document, the ‘Plan national multisectoriel de lutte contre le SIDA et les IST 2014’ [National Multisector Plan for the Fight against AIDS and STDs 2014], stands out from the others – and indeed from the entire corpus – by addressing language issues in a more sustained manner, outlining a total of eight different action points for multilingual HIV/AIDS communication. These encompass translating female condom educational kits ‘en langue nationale’ [into national languages] ( Burkina Faso CNLS, 2014, p. 57); producing guides for children’s religious groups and married couples ‘en français, en arabe et langues nationales’ [in French, Arabic, and national languages] (p. 60); producing educational radio programmes ‘dans les langues locales au niveau des radios communautaires’ [in local languages for community radio] (p. 60); producing ‘en plusieurs langues’ [in several languages] a thrice yearly magazine or comic promoting HIV prevention among adolescents and young people (p. 62); broadcasting a weekly audio and television programme ‘"Parole de jeune pour la santé" en 4 langues’ [Youth Tips for Health in four languages] (p. 62); training HIV advisors in sign language (p. 69); promoting HIV testing through radio and television adverts produced and disseminated ‘en langues nationales (français, mooré, dioula, fulfudé, etc.)’ [in national languages (French, Moore, Dioula, Fulfulde, etc.)] (p. 69). Although these action points give relatively vague details about the languages in which the resources will be made available and indeed do not address the issue of how members of the population who do not have access to those languages will be reached, this plan is the most detailed of all the ones in the corpus in terms of outlining how the multilingual nature of society will be addressed. 

### Ghana Ministry of Health

Three of the four Ghana Ministry of Health documents which mention language do so in the form of a passing mention. Thus the ‘Health Sector Gender Policy’ includes ‘language’ as a factor irrespective of which respect will be shown to individuals ( Ghana Ministry of Health, 2009a); the ‘Occupational Health and Safety Policy and Guidelines for the Health Sector’ state that health and safety information will need to be provided to employees in ‘an appropriate form, taking into account the literacy and the language needs of the employees’ ( Ghana Ministry of Health, 2010, p. 20); and the 2007 ‘Review of Ghana Health Sector’ includes ‘production of IEC materials in local languages’ ( Ghana Ministry of Health, 2008, p. 20) as one of the items featuring in the agenda for action in 2008. The fourth document, the ‘Ghana National Healthcare Quality Strategy 2017–21’ addresses language needs in a more concrete manner, setting specific targets for training health workers in basic sign language ( Ghana Ministry of Health, 2016d, p. 23; 43).

### Ghana Aids Commission

Mentions of sign language are also found in three of the GAC documents: the ‘National HIV Home Based Care Training Manual’ mentions sign language as an example of ‘special cases’ ( Ghana Aids Commission, 2016b, p. 20) with which trainees might deal; a 2016–20 call for expressions of interest from the private sector for involvement in HIV programmes identifies Behaviour Change Communication (BCC) in braille and sign language format as a priority area ( Ghana Aids Commission, 2016c, p. 1); the 2014 ‘Status Report’ notes that IEC/BCC materials were printed in Braille and that sign language was used for audio visuals ( Ghana Aids Commission, 2014, p. 80). The 2014 Status Report also notes that BCC messages ‘were delivered in native languages through drama and poetry for easy appreciation of community members’ ( Ghana Aids Commission, 2014, p. 62), thus flagging up the multilingual aspect of the social contexts in which HIV messages were delivered.

The three other documents which mention language do so in a similarly brief manner. The Global Fund and Ghana Aids Commission joint ‘Request for Proposals RFP/GAC/GF/KP/03/2017’ identifies ‘experience in region and language’ ( The Global Fund and Ghana Aids Commission, 2017, p. 20) as one of the required qualifications and competence of key staff, and also states that it is ‘desirable that the Consultant’s personnel have a working knowledge of the Employer’s national language’ (p. 19). The language issue thus receives limited but nevertheless significant visibility in these proposal request guidelines. The ‘Handbook on Advocacy for Network of Association of Persons Living with HIV and Civil Society Organisations’ has a section on ‘Language’, explaining to readers that ‘language refers to the words you choose to communicate your message. It can also refer to the actual language you use (English, Twi, Ga etc.)’ ( Ghana Aids Commission, 2016a, p. 22). All of the examples that are subsequently provided of how issues of language might play into communication deal however with the intralingual aspect of language, ^5^ meaning that readers receive no guidance on what to do if they find themselves in situations in which more than one actual language is spoken or if the language of the sources in which information is made available does not match the languages understood by the intended recipients. Finally, the ‘National Workplace HIV and AIDS Policy’ states simply that information about HIV/AIDS should be disseminated ‘in a culturally sensitive format and language’ ( Ghana Aids Commission, 2012, p. 20).

### Senegal CNLS

The four Senegal CNLS documents which mention language are all consecutive iterations of the ‘Rapport de situation sur la riposte nationale à l’épidémie de VIH/SIDA Sénégal’ [National Response to the HIV/AIDS Epidemic in Senegal Progress Report] , available on the website for the years 2012–13, 2013–14, 2014–15 and 2015–16. All four reports note an ‘insuffisance des outils de communication en langue locale pour la promotion du CDV’ ( CNLS Senegal, 2014, p. 63) [insufficiency of communication tools in local languages for the promotion of VCT], reproducing this statement verbatim each time. In the first three reports, which are all around 80 pages long, this is the only mention of language. It is only in the fourth report, dated 2015–16, that this problem is matched with a proposed solution: ‘Concevoir, reproduire et diffuser des supports audiovisuels sur les thématiques VIH en langues locales en direction des groupes vulnérables et des groupes passerelles (Femmes enceintes, jeunes, hommes, couple, PS, HSH, CDI…)’ [develop, produce and disseminate audiovisual material on the topic of HIV in local languages targeting key affected populations (pregnant women, youth, men, couples, sex workers, men who have sex with men, people who inject drugs…)] ( CNLS Senegal, 2017, p. 40). The fourth report also introduces the question of the lack of availability of information in sign language ( CNLS Senegal, 2017, p. 61) but does not provide a matching initiative for this perceived deficiency.

## Discussion

The above results show that the extent to which linguistic diversity is taken into account in strategic planning for HIV/AIDS-related health communication is minimal. While 35 of the 314 documents mention language, the level of prominence accorded to language barriers is extremely low: most of the mentions are made in passing or are presented as background socio-cultural information, the implications of which are not explored. Only eight of the documents outline any concrete actions for addressing multilingualism in HIV/AIDS-related health communication. This equates to just 2.5% of documents.

The above results suggest that the likelihood of language being mentioned in policy documents or reports is lowest at donor level and highest at the level of the HIV/AIDS commissions of individual countries. If we combine the individual country results and order the statistics by category, this tendency emerges more clearly, as Table 3 demonstrates.

**Table 3.** Number and percentage of HIV-related policy documents/reports which mention “language” or “translation” in the sense of addressing language barriers. The data was generated by searching the organisations’ official websites between November 2018 and July 2019 and was supplemented by Google searches where websites were not functioning or obviously contained incomplete documentation records. The data is presented by the category of the organisation that produced the document/report.

Category of organisation	Mentions of “language” or “translation” over total number of documents	Percentage of documents which mention “language” or “translation”
Donor ^(a)^	0/67	0 %
International Funding Organisation ^(b)^	14/150	9.33%
National government department (recipient country) ^(c)^	13/52	13.46%
National HIV/AIDS commission (recipient country) ^(c)^	14/45	31.11%

(a) UK Department for International Development (DFID); (b) The Global Fund; (c) Burkina Faso, Ghana, Senegal

While these differences are striking, it is important to remember that the majority of mentions of language are made in passing, with very few documents outlining or reporting on specific plans for addressing language barriers. When reports that do the latter are grouped by category, it is in fact the international funding organisation that comes out on top, as Table 4 shows:

**Table 4.** Number and percentage of HIV-related policy documents/reports which outline concrete plans for addressing language barriers. The data was generated by searching the organisations’ official websites between November 2018 and July 2019 and was supplemented by Google searches where websites were not functioning or obviously contained incomplete documentation records. The data is presented by the category of the organisation that produced the document/report.

Category of organisation	Documents which outline concrete means of addressing language barriers over total number of documents	Percentage of documents which outline concrete means for addressing language barriers
Donor ^(a)^	0/67	0 %
International Funding Organisation ^(b)^	14/150	9.33%
National government department (recipient country) ^(c)^	1/52	1.92%
National HIV/AIDS commission (recipient country) ^(c)^	4/45	8.89%

(a) UK Department for International Development (DFID); (b) The Global Fund; (c) Burkina Faso, Ghana, Senegal

The limited number of countries and organisations included in our study make it difficult, however, to draw anything more than tentative conclusions about the organisational levels at which multilingualism is taken into account. Had we selected a different international funding organisation, for example, it is possible that the figures would look rather different. The same goes for the selection of countries: Senegal, with its very few mentions of language, brings the percentages for the recipient country categories down. It is possible that the statistics would look rather different had we chosen another country in its stead.

It is also important to note that the strategic plans and reports published on the organisations’ websites do not tell the whole story. While the Senegal CNLS website contains only one document that outlines specific plans for addressing language barriers, for example, the media library tab on the website contains documentaries and adverts in Senegal’s most widely spoken national language, Wolof, as well as the official language, French ^6^. From this and other evidence, it is clear that formal health communication activities (in addition to informal, ad-hoc communication between health professionals and patients) do take place in a range of languages in all three of the countries on which we focussed, but their visibility in formal planning and reporting is extremely low.

Many of the outlines of multilingual initiatives are phrased in general terms and thus make it impossible to ascertain the precise nature of the activities that are envisaged or have been undertaken. For example, the Global Fund technical brief ‘Addressing Sex Workers, Men who have Sex with Men, Transgender People…’ states that funding requests should ensure that ‘the content of behavioral interventions, and of material published in print or online, should be adapted to take into account the needs, culture and language of the key population in question’ ( The Global Fund, 2017, p. 12). The document leaves open the way in which content and material might be adapted and assumes through its wording that the ‘key population’ shares a single culture and language.

Where precise activities can be identified in the corpus, they fall into two categories:

1.    Training of health care workers. This strategy is mentioned only in connection with sign language. There is no report of any plans to encourage health workers to learn other national languages. This is surprising in the sense that this is a solution that, anecdotally at least, is often adopted by health professionals who find themselves working primarily with patients who speak a language that they do not know; furthermore, it is also a solution that a number of researchers have highlighted as being among the most effective in ensuring effective communication that is built on trust ( Claasen *et al.*, 2017).
2.    Large scale sensitization campaigns through the production and dissemination of radio or television programmes or adverts in more than one language. While some documents do mention plans for producing written material, in the majority of cases the preferred channel for large-scale communication is audiovisual. This can no doubt be explained by taking into consideration the literacy rates in the three countries under study as well as in many of the countries targeted by DFID or the Global Fund. As we saw above, the conflation of literacy with language proficiency is however not always helpful for thinking through ways to make communication maximally effective.

It is notable that none of the specific initiatives for addressing language barriers envisage making use of internet- or mobile phone-based platforms. This is perhaps a reflection of the lack of sustainability and inclusivity of such initiatives in countries where electricity provision is unreliable and high-speed internet access is limited to city centres ^7^, but it is also the case that such initiatives may be going under-reported: in Ghana, for example, there is documented use of teleconsultations by Kumasi Hospital ( Molefi, 2010), and other e- and m-health initiatives have been proposed ( Osae-Larbi, 2016).

As indicated above, the silence on language barriers that is a feature of the corpus is not matched by a silence on communication in general. For example, the Ghana Ministry of Health’s ‘Review of Community-based Health Planning Services’ makes no mention of language, but mentions communication 13 times ( Ghana Ministry of Health, 2009b). Similarly, there is an emphasis throughout the corpus on the importance of community-based approaches and on the role played by community workers and volunteers. The DFID report ‘Towards Zero Infections: Two Years On’, for example, states that the Community-led TB-HIV Advocacy in Zambia (COTHAZ) project ‘trained 100 volunteers […] to provide outreach visits to local communities. Through communicating accurate information, they are challenging myths and stigma surrounding both illnesses’ ( Department for International Development, 2013, p. 33). It is undoubtedly the case that a key part of community volunteers’ importance and their ability to communicate ‘accurate information’ lies in their language competence: in very simple terms, such volunteers speak the same language as the people in their communities and thus do not face this considerable barrier to effective and accurate communication. It is of course also the case that community volunteers understand and know how to address the culturally-based concerns and preconceptions that might have an effect on the understanding and reception of HIV/AIDS-related information and advice.

The tendency to speak of ‘communication’ and ‘community’ rather than to evoke the need for specifically multilingual or language-sensitive approaches might be deemed to be of little importance. After all, it could be argued that policy makers and implementing partners within African countries or other LMICs would be aware of the linguistic make-up of their own countries and therefore not need to spell out the necessity of communicating in a wide range of languages. However, it is almost certainly the case that if language needs are not visible within the funding chain, then they will not have sufficient resources dedicated to them. This, in any case, would be a hypothesis that needs testing, and one which has far-reaching ramifications for the way in which HIV/AIDS-related health funding is allocated.

This hypothesis thus highlights the need for further research in this area. It is not only the findings of this study that need further verification against larger bodies of data and a greater range of organisations and government departments, but it is also crucial that desk-based surveys like the present one be combined with studies of the ways in which health communication initiatives are actually implemented in multilingual societies. Such combined studies will allow us to better understand the impact of the low visibility of language issues on the effectiveness of health communication, particularly in parts of the world that are linguistically diverse or where languages exist in a hegemonic relationship to each other. As noted above, these questions are of keen importance for gender equality: while it has long been recognised that education is crucial in improving the lives and health of women and girls, what has often been overlooked is that one of the key skills that is acquired through education in many linguistically diverse countries is the official language of the country in which girls live. This language allows women and girls to access health care and information within their own countries; if it is a global language, then it gives them access to a large body of health-related information; if it is English, the lingua franca of scientific research and the language in which most of the world’s public health information is provided, then it potentially allows women and girls to cross the divide between the ‘health-information haves and have-nots’, to use the words of a World Health Organisation report ( Adams & Fleck, 2015) ^8^.

## Data availability

### Underlying data

All data underlying the results are available as part of the article and no additional source data are required.

### Extended data

University College London data repository: Supporting Data for Multilingualism and Strategic Planning, Batchelor *et al.* (Table 5). https://doi.org/10.5522/04/10738787 ( Batchelor *et al.*, 2019)

This project contains the following extended data:

• Batchelor Multilingualism and Strategic Planning Supporting Data (Table 5).pdf (Table of all reviewed documents)

Data are available under the terms of the Creative Commons Zero "No rights reserved" data waiver (CC0 1.0 Public domain dedication).

## Footnotes

^1^ Described by Greenberg (1956, p. 109) as follows: ‘If from a given area we choose two members of the population at random, the probability that these two individuals speaks the same language’.

^2^ Prah (2011), for example, argues that the multilingualism of the continent is overstated and identifies 15–17 core languages that account for 85% of the speakers on the continent. For a more general problematisation of the calculation of linguistic diversity, see Skutnabb-Kangas & Harmon (2018).

^3^ According to the 2017 Statistics on International Development report, Africa receives the largest percentage share of UK bilateral ODA allocated to a specific region (around 50% of bilateral ODA, which itself accounts for around two-thirds of the total spend) ( Department for International Development, 2018b).

^4^ DFID’s 2018 Single Departmental Plan, for example, states that DFID will ‘continue to seek to reform multilateral institutions, especially in the way they distribute development funds’ ( Department for International Development, 2018a, p. n.p.).

^5^ The Handbook asks the readers to consider the following intralingual issues: • Is the word choice clear, or could various audiences interpret it differently? • Is the language appropriate for your target audience? Obviously you would use a different language when appealing to university researchers than you would when communicating to a youth group. Be careful not to use offensive words. • Use words that conjure up images and raise emotions, such as empathy for suffering children • Use familiar words the audience will understand and identify with’ ( Ghana Aids Commission, 2016a, p. 22)

^6^ See https://www.cnls-senegal.org/mediatheque/films-documentaires/ and https://www.cnls-senegal.org/mediatheque/spots/ .

^7^ For an overview of social media for health communication in Africa, see Fayoyin, 2016.

^8^ We should note, however, that many of them will still be disadvantaged by the exacerbating factors noted in the same report, namely poor internet connectivity and unreliable electrical supplies.
