# Supplementary material for: Multilingualism and strategic planning for HIV/AIDS-related health care and communication
Source: Wellcome Open Res. 2019 Dec 12;4:200. [Version 1] doi: 10.12688/wellcomeopenres.15584.1 (PMC6948712; doi:10.12688/wellcomeopenres.15584.1)
Supplement: Peer review history

# Peer review: Reviewer response for version 1

**DOI:** 10.21956/wellcomeopenres.17066.r37381

**Published:** 2020-01-17

**Authors:** Kobus Marais

**Affiliations:** 1 University of the Free State, Bloemfontein, South Africa

The paper studies existing documents from West-African countries on HIV/AIDS that relate to interlingual translation. It collected data from four categories of institutions and tried to ascertain whether these documents mention interlingual translation as an issue in health-care communication. The empirical investigation is set within a theoretical framework that is sensitive to the context in which the research is done, focusing on development issues. The findings are in line with the research problem and flows from the data presented. The ways in which the data was analysed are clear and transparent.

 I made a number of minor comments in the text:

1. How do you use the term 'West' Africa? Is it just generally geographic, or do are you talking about ECOWAS? Clarifying this might help the reader to understand the context.
2. Introduction, paragraph 6: Also see Chibamba (2018) ^1^. 
3. Analysis of documents, paragraph 2: I find this statement problematic. Jakobson made clear that interlingual translation is not the only activity worthy of the name 'translation'. He also included interlinguistic and intersemiotic translation, so I would suggest you reformulate this sentence.
4. Later in same paragraph: I think we should indeed study all of these as translation. That said, I understand that you need to hedge your choice of data.
5. References: I am missing references to recent work by a number of scholars such as Footitt, Teseur, Delgado Luchner and even myself. While these are not specifically about health care, the first three do reflect on interlingual translation in the chains of development projects. It might be to the advantage of the authors to take note of these, but it is probably not crucial.

That said, I think this is an excellent paper, written with insight and sensitivity, and making a great contribution to translation studies.

I confirm that I have read this submission and believe that I have an appropriate level of expertise to confirm that it is of an acceptable scientific standard.

## References

[1] : Translation Practices in a Developmental Context: An Exploration of Public Health Communication in Zambia. *PhD Thesis, University of Ottawa*.2018;

# Peer review: Reviewer response for version 1

**DOI:** 10.21956/wellcomeopenres.17066.r37383

**Published:** 2020-01-07

**Authors:** Carmen Delgado Luchner

**Affiliations:** 1 Global Studies Institute (GSI), University of Geneva, Geneva, Switzerland

This article tackles a highly relevant and hitherto under-researched issue, namely the prominence of issues of language and translation in the planning of HIV/AIDS-related communication in three highly multilingual West African countries, Burkina Faso, Ghana and Senegal. Based on their analysis of the existing literature on language barriers in health care and the linguistic diversity of African societies, the authors posit that health-related communication in West Africa needs to be provided in several languages and/or involve translation in order to reach the populations affected by HIV/AIDS.

 In the empirical part of the paper, the authors present a qualitative and quantitative analysis of HIV/AIDS-related policy documents and reports in order to assess the importance given to linguistic diversity. The research design involves three country case studies, each supported by a diverse corpus of documents combining national and international sources.

 The findings suggest that minimal attention is given to linguistic diversity across the board, with only 2.5% of the examined documents containing any reference to concrete actions for addressing multilingualism in HIV/AIDS-related health communication. This study thus points to a mismatch between the prevalence of multilingualism on the ground and its lack of visibility at planning and policy level, which has also been documented in other settings, namely in development NGOs and humanitarian organizations.

 The papers strength resides in its systematic approach, and its attempt to quantify, document and describe a phenomenon that has not yet been studied in depth. The authors disclose the limitations of their study, namely the fact that a different selection of cases might have produced different findings. In this regard, it would have been interesting to provide some information as to why three countries with a comparatively low prevalence of HIV/AIDS were chosen, since a higher prevalence might raise the stakes of HIV/AIDS-related communication.

 The study's tentative conclusions are thought-provoking and raise numerous questions that could be explored in subsequent studies. As they move forward in their research, I would encourage the authors to fully engage with the methodological and conceptual implications of the 'predominantly oral' nature of communication in local languages. Indeed, framing multilingualism mainly or predominantly in terms of 'translation' might lead scholars to overlook the myriad hybrid communication practices that exist in African settings, including the wide-spread use of pictographs accompanied by oral explanations in the local language to provide health-care information to illiterate audiences. This aspect could also be explored indirectly on the existing data set by probing for terms such as "sensitization" or "awareness-raising" but also "vocabulary" and "terminology", which in my experience in researching the multilingual communication practices of NGOs are often used as a proxy to refer to settings that involve oral interpretation, sight translation, code-mixing and similar multilingual practices.

I confirm that I have read this submission and believe that I have an appropriate level of expertise to confirm that it is of an acceptable scientific standard.

# Response

**Published:** 2020-01-14

**Authors:** Kathryn Batchelor

**Affiliations:** University College London, UK

We thank the reviewer for her positive comments and for carrying out the review so quickly. The reviewer highlights the importance of the article’s findings and notes that it correlates with findings in related studies. The reviewer wonders why we did not select countries with higher HIV/AIDS prevalence rates. We agree that such a study would offer an extremely useful comparison. The current study is however part of a broader effort to study health communication and multilingualism in the three selected countries, and will be complemented by field-based research. We thank the reviewer for her extremely insightful and useful suggestions for further research directions and will make full use of these as we develop our research further.

# Peer review: Reviewer response for version 1

**DOI:** 10.21956/wellcomeopenres.17066.r37382

**Published:** 2019-12-20

**Authors:** Sharon O'Brien

**Affiliations:** 1 Dublin City University, Dublin, Ireland

This article tackles the question of how multilingual access to health information, through translation, is established in policy and guidance documents in a West African context.

 The complex problem of access to multilingual health communication in an African context is set out well, highlighting issues such as how to measure ‘linguistic diversity’, and taking into account the fact that African multilingualism is predominantly oral, sometimes accompanied by very limited literacy levels and an absence of agreed terminology. In addition, the linked aspects of education and gender are briefly presented.

 The lack of research in the African context is also highlighted, demonstrating that efforts to date are restricted to specific geographic regions (South Africa) or to specific health issues (Ebola). The need for a broader examination of the issue is thus clearly established.

 The main research aim is well articulated: assessing the extent to which linguistic diversity is considered for strategic planning for HIV/AIDS health care and communication, with a particular focus on West Africa. The justification given for the inclusion of organisations and their related documents is convincing. While internet searches cannot guarantee that all relevant documents have been located, the description of how the systematic search was carried out is transparent and resulted in a significant number of relevant documents for analysis (314, with only 11% of these, however, containing relevant content). The authors demonstrate an awareness of the limitations in the scope of their analysis. Given how little attention the topic has received to date, the analysis is nonetheless a good contribution to the topic.

 The results of the analysis are, unfortunately, unsurprising, given that similar work in the area of crisis response and the development sector has also found a very limited recognition of the need for translation in policy and guidance documents. Where language and translation are mentioned in the corpus, these mentions are often cursory and/or repetitive, as demonstrated by the detailed analysis of the documents under investigation.

 The lack of consideration of multilingual communication is rather shocking when the context of multilingualism and (sometimes) low literacy is considered. This is symptomatic of a much larger blind spot on the importance of language and translation across governmental organisations and NGOs internationally. As highlighted by the authors, the lack of mention of language is problematic in itself, but lack of mention in policy and guidance documents also implies a lack of action, which is of a much greater concern, given the potential ramifications.

 The authors call for more research on the topic, but also rightly point out that the need really lies with investigating the actual implementation (and the success or failure) of communication campaigns on the ground in highly diverse multilingual settings. While a call for more research, and especially field research, is welcome, the authors might have gone a few steps further by making concrete policy recommendations for inclusion in the types of documents they analysed.

 There were a few minor aspects of the article that could be improved. For example:

 In the Introduction, para. 4, a statement is made that when healthcare information is provided in a single language this is “predominantly English or French”. This statement needs to be supported by referring exactly to which parts of the globe this pertains.

 On page 4, where the justification is given for the inclusion of specific Ministries of Health, it would be useful to have a footnote providing an explanation of what the linguistic diversity indices typically mean.

 It was a little surprising that “interpreting” and derivations of that term were not listed as a search term, given the role of interpreting in health communication and the point made about the dominance of orality in the African context. It would be useful if the authors addressed this issue. Presumably, on close reading of the documents, it was found that there were no occurrences of such terms?

I confirm that I have read this submission and believe that I have an appropriate level of expertise to confirm that it is of an acceptable scientific standard.

# Response

**Published:** 2020-01-14

**Authors:** Kathryn Batchelor

**Affiliations:** University College London, UK

We thank the reviewer for her positive and constructive comments on the article, and for carrying out the review so swiftly. The reviewer highlights the importance of the research, commenting that the findings are in line with those of similar studies carried out in crisis settings and the development sector. The reviewer recommends that the article might have made concrete policy recommendations. While we agree that changes to policy are urgently needed, we plan to make such recommendations at a slightly later stage, based not only on this article but also on field research. Our responses to the specific issues highlighted by the reviewer are as follows:

**Introduction, para 4, English and French use in Africa:**

 The goal of this section is to give readers unfamiliar with the linguistic situation in Africa some points of orientation, rather than to provide a more complete overview, and we are keen to keep it succinct. As the validity of the statement as phrased does hold (the context makes clear that this statement refers to Africa rather than other parts of the globe), we have decided to leave it as it is.

**Page 4, linguistic diversity:**

 A definition of linguistic diversity is given earlier in the article. We prefer to keep the statement on page 4 as it is rather than explaining how this might correlate to numbers of languages, and refer the reader to the reference provided (Ethnologue) for further information.

**Absence of ‘interpreting’ from search terms**

 The reviewer comments on the absence of ‘interpreting’ and its variations from the search terms used to interrogate the documents. We thank the reviewer for this pertinent point and confirm that close readings of the documents did not find any hits for ‘interpreting’ or its variants. We also note that although researchers and those working in the translation professions are careful to observe the distinction between ‘translation’ (written) and ‘interpreting’ (oral), these terms tend to be used interchangeably in popular discourse in both English and French, with ‘translation’ being the preferred term.

# Peer review: Reviewer response for version 1

**DOI:** 10.21956/wellcomeopenres.17066.r37380

**Published:** 2019-12-19

**Authors:** Hilary Footitt

**Affiliations:** 1 University of Reading, Reading, UK

This article tackles a vital but hitherto less researched area of healthcare, namely the role of languages in the strategic planning of health interventions. The authors start from the observation that research on migrant community health care has indicated that language barriers can have an adverse impact on health care, affecting both access to care, and the quality of care received. They point out that whilst intralingual communication remains a key focus of practitioner training, the problematics of interlingual communication in non-Western health interventions have seldom been explored. In African contexts for example, such language barriers are particularly acute, given the wide range of languages spoken, the often oral nature of African multilingualism, and the fact that the language designated as official (often English or French) is actually understood by only a minority of the population, and often not by women who are disproportionately affected by HIV/AIDS in the Sub-Saharan African region. The article proposes to explore the extent to which linguistic diversity is taken into account in the strategic planning for HIV/AIDS health care in West African contexts, concentrating on Burkina Faso, Ghana and Senegal.

 Methodologically, the article uses quantitative and qualitative analysis of HIV/AIDS-related documents and reports from the websites of donors (DFID), funders (The Global Fund), and the Ministries of Health and National Aids Commissions in the three countries. The corpus of 314 documents is interrogated to ascertain whether linguistic diversity is noted as a factor in strategic planning, and the level at which such planning takes place. The authors clearly set out the ways in which the documents were selected in each case, and acknowledge the limitations of their research.

 Their conclusions are striking. They conclude that the extent to which linguistic diversity is taken into account in strategic planning is minimal, with a suggestion that the likelihood of language being mentioned is lowest at donor level, and highest at the level of in-country commissions. The silence on language barriers which they detect is not matched by a similar silence in these documents on communication in general, or on the desirability of community-based approaches. Whilst it might be argued that partners and policy-makers within the African countries can be left to address the diversity of language needs, this apparent lack of any overt recognition of multilingualism throughout the policy-making chain suggests that the resources needed to translate and interpret in these highly diverse linguistic contexts are very unlikely to be provided.

 Overall, my only suggestion which the authors might consider is to strengthen the initial part of the article with a slightly fuller description of current work on the problematics of migrant communities assessing health care in Western settings where conclusions on access, quality of care, and costs associated with languages provide helpful pointers for the article’s broader considerations of multilingualism and health care in the African context.

 This is an original and carefully written article which strongly points to the need for further research on the ways in which health initiatives are actually implemented in multilingual societies, how the actions of health policies are actually translated into the words of the receiving communities.

I confirm that I have read this submission and believe that I have an appropriate level of expertise to confirm that it is of an acceptable scientific standard.

# Response

**Published:** 2020-01-14

**Authors:** Kathryn Batchelor

**Affiliations:** University College London, UK

We thank the reviewer for her detailed and positive comments and for reviewing the article so swiftly. The reviewer highlights the vital importance of language issues in health care planning and the pressing need for further research in this area. We note the reviewer's suggestion that the introductory section of the article might have benefited from a more detailed description of existing work on migrants' access to health care in the West. As the article is already rather long, we do not propose to expand it, but we agree that the findings of researchers working in that closely related area form an important backdrop to our research.
